# Reproductive medicine in northwest Argentina: traditional and institutional systems

**DOI:** 10.1186/1746-4269-3-19

**Published:** 2007-05-02

**Authors:** Norma I Hilgert, Guillermo E Gil

**Affiliations:** 1 Centro de Investigaciones del Bosque Atlántico, Universidad Nacional de Misiones, CONICET. Casilla de Correo 8 (3370) Puerto Iguazú, Misiones, Argentina; 2 Administración de Parques Nacionales, Argentina

## Abstract

**Background:**

The state of conservation of the traditional cultures of Northwest Argentina is variable and somewhat problematic but to a lesser or a greater extent all the peoples are related to an hegemonic culture. We present a case study carried out in the rural communities of the Yungas biome (Salta) where the extent of isolation varies as does the type of access to public health services. The use of medicinal plants in the area is ordinary and widely spread.

**Methods:**

The data can be organized in two categories, as medical systems public records (for the regional hospital at Los Toldos), and as ethnobotanical sets. A total of 59 surveys to 40 interviewees were undertaken using a semi structured questionnaire. We present an analysis of the relative importance of the medicinal herbs used in reproductive medicine considering the plants used in the traditional medical system and the factors that can affect the relationship between formal medicine and patients. We further analized how the degree of accessibility to the local hospital influences the diversity of use of plant species used to assist deliveries and to decrease infant mortality in children minor than one year of age.

**Results:**

In reproductive medicine, 13 ailments and/or different physiological states are locally identified and treated. Local population uses 108 ethnospecies for this kind of illnesses. According to the local conception the hot/cold imbalance could be the principal cause for reproductive illnesses; pregnancy may have natural or supernatural origin, post partum and menstruation involve similar sanitary risks, and neonatal care has a strong magic connotation. In relation with the formal medicine, the more accessible is the health center the more women assist to it. We have not found a relation between accessibility and infant mortality.

**Conclusion:**

In the local reproductive medicine, most of the practices are concerned with the hot/cold balance. According to their importance the factors involved are: the family medicine, the midwife, and the formal doctors. Plants have an important role; however there is a lack of total agreement among the families who use them. Reluctance to institutional deliveries may be due to the weak relationship between patients and doctors, and the lack of logistic assistance to delivering mothers coming from far away locations.

## Background

The Andean medical system is formed by a complex combination of herbal knowledge, magic elements and propitiatory rituals. The general cultural picture were described as a true tapestry of folk practices where the elements proper of the region and those that have been introduced by migrations both in pre-Columbian times as well as after the arrival of the Spanish are difficult to sort out [1]. As a result there are numerous and even contradictory interpretations of the local cultural lore. Clear examples of this old dilemma can be found in the analysis of the hot/cold concept origin or of affections such as the "air" and "evil eye" [2-8].

There are numerous studies on medicine and medicinal plants in the Argentine northwest; most were carried out in the desertic or semidesertic environments of the Puna and Prepuna. Although the bioclimatical differences between these places and the cloud forest where we conducted our study are important, it is acceptable in general terms to consider the traditional medicine of the region as a cultural unit, probably due to the frequent communication among the communities and to the ancient regional influence of the herbal tradition of the Kallawayas [5,9]. Numerous studies on the cultural aspects and the plants used in female medicine – gynecological affections, deliveries and birth rate control, have been made in America [10-17].

Different studies made in the Andean region of Argentina have agreed on the importance of certain ritual and magical practices, besides medicinal plants and culinary elements, in the Andean reproductive life [i.e. [5,18-22]]. Some examples are the burial of the placenta and annexes as an offering to the Pachamama (a ctonic goddess of the Andean world) and the use of "hilo yoque" or "lloq'e" – a cord of white and black wool weaved to the left, with ritual value, used to tie the umbilical cord, among others.

The observance of strict cultural rules for personal feeding during pregnancy, after the delivery and during breast feeding have been recorded in different regions of America in communities with Quichuan influence. In general, special meals are eaten to counter the temperature imbalance that the embryo generates during the gestation and birth, to maintain the necessary hot temperature for adequate milk production or to avoid the "cooling" of the uterus to ensure its future fertility [15,19,23,24]. The study of food with therapeutic effects in different regions and ethnic groups has revealed its particular importance and on numerous occasions it has been underestimated when analyzing local medical systems [25-27].

Another important characteristic in the Andean communities is the role of domestic medicine; in most cases, relatives – usually mothers – are primary health caretakers [5,15,18,19,24,28,29]. It has been observed that local decisions to involve the official medical system are influenced by values that go beyond the mere availability or access to the health centers or hospitals [28].

In this study we present an analysis of the reproductive ailments known and treated whitin the traditional health system, the cultural values which affect treatments, the herbs used and their relative importance, and the factors which can affect the relationship between the formal medicine and the patients. We also adopt a primary approach to the elements that govern the relationship between the people in charge of the health centers and the delivering mothers. We also analyze the extent to which the accessibility to a health centre influences the diversity of use of the species used for the assistance of the deliveries and to decrease mortality of children under one year.

We have considered as reproductive medicine all the practices involved in the conception, pregnancy and delivery, the care of newly born babies and the treatment of female diseases which affect reproductive success.

### Study area, Socio-cultural and Ethnomedical traits

**Figure 1:**
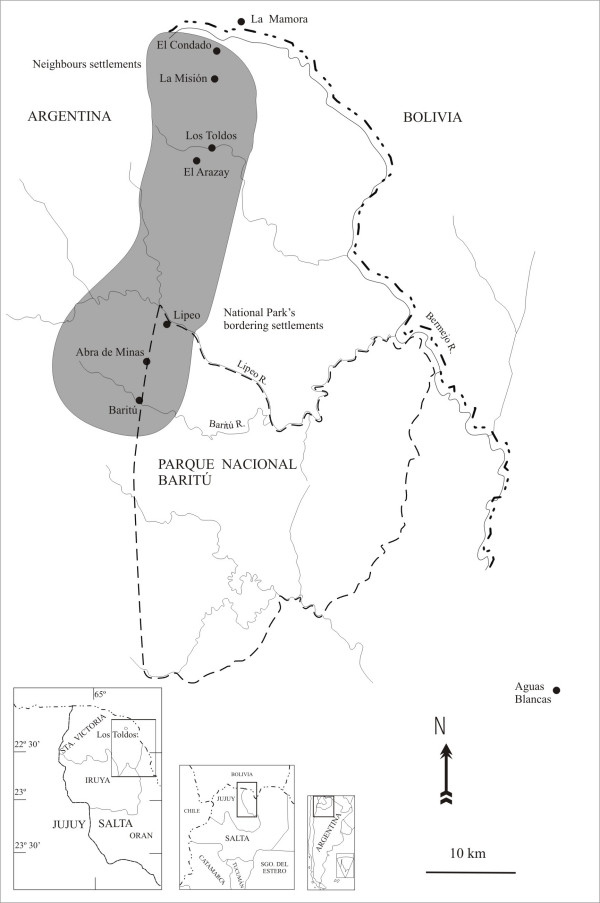
Study Area.

This work was carried out in the Upper Bermejo Basin, which is located near the Bolivian border to the north of the province of Salta, Santa Victoria district (64° 45' W and 22° 25' S) (Fig. 1). The localities are placed at 1,100 and 1,500 mosl, on water streams of the Bermejo river basin. Cabrera and Willink include this area in the biogeographic province of the Yungas (or mountane moist forests) (Neotropical Region, Amazonic domain) [30]. It is possible to distinguish two different environments in this region: the montane subtropical forest and the temperate cloud forest [31]. The composition and floristic elements of these units have been described in Cabrera and Hueck [31,32]. The climate is tropical continental, with warm rainy summers, and cold dry winters. The annual mean temperatures oscillate between 14°C and 26.5°C. Rainfall is centered between November and March and varies between 700 and 1,400 mm. per annum [33].

The settlements are living in a subsistence economy based on shifting agriculture and intensive cattle breeding. They are considered part of the Andean cultural world. The inhabitants of these settlements are descendants from the original Quechuan communities with a strong Spanish influence; they speak only Spanish, although many Quechuan expressions are found in their speech. A brief analysis of the regional economy shows the coexistence of shifting agriculture, migration, gathering, nomadic cattlebreeding, hunting and fishing, and finally, access to paid work in temporary or permanent jobs. More details about key socio-economic and environmental features of each study area can be found in Hilgert and Gil [28].

The inhabitants of the region practice traditional medicine. We understand as traditional medicine the people's present local lore, it is to say we agree with that stated by Menéndez [34] and Bhatti [35], we consider that the traditional lore is a permanent recreation on the part of the individuals and communities to confront the challenges posed by the social and cultural environments.

In this local system the underlying notion of the origin of a disease is very important to determine its diagnosis and treatment. Diseases are attributed to one of three sources: natural, sociocultural, and supernatural. The diseases of natural origin are usually treated within the family context with the use of different elements of the natural pharmacopeias without including the use of any prayers or rituals. The rest of the diseases are diagnosed by the rural doctor who will indicate if he can provide a treatment or if the patient should go to the health center. The rural doctor is consulted also if an ailment that has been treated within the domestic domain does not get better or presents complications (in this case it is suspected that a mistake was made either in the identification of the etiology or in the measures taken). In this region both the rural doctor (who is a specialist in natural pharmacopoeia) and the curandero (who is a specialist in treatments generally derived from bad air or witchcraft) are known as "rural doctors". It is common that these two people work together according to the complexity of the case.

The hot and cold theory holds an important place in the local medicine lore. Its conceptual basis coincides mainly with that already proposed by several authors [5,15,24,28,36-38]. The local medicinal lore considers a healthy body to have a certain corporal temperature that can be altered by the influence of external factors, e.g., wind or sun, or as a result of eating "hot" or "cold" food in excess. Cold and hot diseases are generally considered to be of natural origin. Apart from being illnesses themselves, they can promote other illnesses. As the information on the aforementioned etiological characteristics and adequate treatment for a number of ailments caused as a consequence of templary complications as well as all of those with a non natural origin, exceed the people's cognitive domain, and is concentrated in the rural doctor, no more details about them are included in the present study.

## Materials and methods

### Data collection

The data can be organized in two categories, as ethnobotanical sets and as medical systems public records. The ethnobotanical data were collected in two steps. First, along 7 visits from 1994 to 2000 global ethnobotanical practices of local medical knowledge were summarized, and during 4 visits from 2000 to 2001 59 surveys to 40 interviewees were undertaken using a semi structured questionnaire. In the first step the semi structured questionnaire was directly interested in the global local medicine [28], and afterwards specifically in the reproductive one. The individuals were asked about the medicinal plants they used, the parts of the plant used, the methods of preparation and administration, the dosage, the duration of the treatment and the illness being treated. Plants were collected with local help and were pressed and dried at the Museo de Ciencias Naturales of the Universidad Nacional de Salta, Argentina. The nomenclature used follows Zuloaga et al. [39-41].

The Medical System Archives were consulted at the end of 2000 in the regional hospital in Los Toldos. There, the available information had different levels of details for each community according to the effort invested by each sanitary agent and the availability of the documents. But, in all cases the same synthesis file was available, which provided us comparative information.

### Data analysis

The relative importance (RI) for each species was estimated based on the normalized number of pharmacological properties attributed to each of them and the normalized number of ailments treated with each one, following the protocol of Bennet and Prance [42] with some modifications. To analyse the similarities or differences in the way of use of the plants among the communities, two types of approaches were made. First, we compared the group of communities based on the quantity of informants by ethno-species using the Kruskal-Wallis' non-parametric test with paired data ranks. Afterwards, to detect specifically which pairs of settlements showed a significant difference, Nemeyi's multiple comparison test was applied with a different quantity of data in each group and paired data ranks. The other aspect analysed was the diversity of ailments and therapeutic actions in which each ethno-species is used and its comparison between settlements. To do this, the unit of analysis was the quantity of informants which reported uses by ailment for the 28 etno-species with higher IR (≥20). The Shannon-Wiener's diversity index was calculated using the natural logarithm (with *e *base) in its formula and the two-tail *t *test, for the comparison between pairs of settlements. All these analyses were done using Microsoft^® ^Excel Office XP (1985–2001 Microsoft Corporation) spreadsheets and formulas and tables from Zar [43]. We also analysed the degree of association among the accessibility to the health centre and the mortality of children under one year, and assisted or non-assisted deliveries. In order to do this, the communities were registered according to their degree of accessibility taking into account: the distance, the existence of regular public transport and road conditions along the year. The factors which facilitate the access to the Health Centre were taken into account because besides fostering regular visits, they influence the ability of doctors to create a confident relationship with the patients. Even though all communities have a sanitary post, doctors are rarely present in them. The sanitary posts have permanent or semi-permanent assistance of a nurse or a sanitary agent, in charge of primary assistance and general sanitary control of the families, respectively. With this method we defined 4 groups according to their decreasing degree of accessibility:1) Los Toldos, 2) La Misión and El Arazay, 3) El Condado and 4) Lipeo and Baritú; absolute values – of in-hospital births and mortality of children under one year – were transformed to percentages of the total by community. With these ordinal values Spearman's correlations were made with the Statistica program [44].

## Results

The information has been organized as follows: first we describe the characteristics of the institutional system. Then we point out the aspects associated to reproductive medicine in the local health system: family planning, reproduction and gynaecological conditions, from the emic perspective. Finally, contraceptive practices and abortion, pregnancy, delivery, post partum and their possible complications (air, "inflamación" and "desmagradura" or "lastimadura"), care of the newborn and female aliments (menstrual pains, "margarita*s*" and "sentaderas") are analysed.

On the other hand, we present the results about the plants used, from an ethnobotanical perspective, the use of edible species with preventive or curative purposes and finally the relation that the mothers maintain with both medical systems, the traditional and the institutional one, in what respects to reproductive medicine.

### The institutional health systems

The institutional health system is provided by the Health Secretary which depends from the National Health Ministry. In the city of San Ramón de la Nueva Orán there is a central hospital for all the Northeast of the Province of Salta. There, the activities of the Program for Health Primary Assistance for the region are coordinated. The cases with medium to high complications that cannot be solved in the periphery Health Centres are derived to this hospital. If the problem exceeds the operative capacity of this hospital the patient is derived to the City of Salta, head of the province.

Los Toldos Health Centre which depends form a previous one located in the same community is located in the study area; locally it is known as "the hospital". At the time we carried out our field work, the centre had two resident doctors, a biochemist, and infirmary and social work assistants. It had a shared ward and it did count with any infrastructure for the accommodation of any relatives accompanying the patients who came from distant locations. Besides no board service were provided during the patient's stay at the hospital, it was the responsibility of the relatives to bring the patients' food.

Apart from this centre there are sanitary posts in each of the studied settlements, there is usually a nurse or a social worker assisting the people. The first is in charge of assisting minor ailments in situ or derive the patient to Los Toldos; she/he makes sanitary check-ups for pregnant women and children and keeps a record of the vaccination calendar for each family. The sanitary agent is in charge of controlling the weight and nutritional condition of children and elderly inhabitants periodically, providing powder milk to families with nursing children, controlling peridomestic hygiene, and suggesting or advising on the construction of latrines.

### The local health system: family planning, reproduction and gynecological conditions

#### Contraceptive practices and abortion

Due to the local practices it is difficult to analyse these two aspects separately. In exceptional cases the use of plants to prevent pregnancy was registered (see Additional file 1), some with a temporary and others with a permanent effect. Most of the settlers are Catholic while the rest have recently converted to Evangelist sects. Special prayers performed to avoid pregnancy are considered as the best method to prevent pregnancy. Unwanted pregnancies are perceived to be the result of not being sufficiently devout to God.

Some plants considered as contraceptives, according to the moment of the menstrual cycle in which they are used, actually seem to have an abortive effect. In these cases the infusion is drunk two or three days before the probable date of menstruation.

The use of plants to induce abortion is common. In the local lore it is considered that if taking this type of medication successfully induces abortion, the pregnancy came from an unnoticed evil seduction or a supernatural agent. On the other hand if the pregnancy fails to terminate, the baby is considered a man's son and so it belongs to God.

It is habitual that during a talk a mother makes reference to one of her children and add "this one I didn't want to have, that's why he is so black, he was cooked with the hot herbs I took to expel him". Abortive plants are considered hot, and they are thought to cook the embryo thus stopping gestation.

The occurrence of spontaneous abortion is explained in the region as a consequence of a unfulfilled wish. A woman is said to be "wishful" when she loses a baby. For instance if a pregnant woman sees a specific food, or the preparation of a meal and she is not invited to share it, abortion can occur as the baby has not been satisfied. To avoid this unfortunate ending, it is usual to encourage pregnant women to express all their wishes, and whenever possible to fulfill them.

### Pregnancy

This period is considered particularly risky for the mother's life, an informant said that "during pregnancy one has one foot in the grave, because one has the *madre *(uterus) open and the earth can swallow you". The baby is able to produce an important temperature imbalance that can put the mother at risk of getting *air *during the delivery. This illness could cause her mental disorders or even death. So, during the pregnancy it is necessary to observe measures in feeding which will be explained below.

Another precaution that expectant mothers have to take is not to attend wakes or burials, and not to go to the cemetery. It is consider that the soul of the death could affect the baby who could later get an illness known as "aicadura", which causes general deterioration, strong diarrhea and death as a result of malnutrition.

Sexual intercourse is not prohibited during most of the pregnancy, but it's forbidden to nurse during gestation, it is said that the fetus will cause the nursing baby to be ill.

### Delivery

Generally, a week before the estimated date of birth the expectant mother bathes with fresh herbs. This is done with the purpose of "taking out" all the warmth accumulated in her belly as a result of the baby and of the daily proximity to the cooking stove.

As explained above it is thought that the baby increases his mother's body warmth, thus at the moment of birth it is imperative to prevent a sudden "cooling". For that reason, when labour starts, it is important to put on very warm clothes (woolen if possible). If the mother catches a cold or drinks water that has not been boiled, it is said that her blood freezes and stays inside, which causes severe pain and will make it difficult for her to get back to her previous body shape, that is why she should only drink boiled water or infusions.

Aromatic herbs are fumigated during the delivery to chase away the bad spirits that could enter the body through the vaginal channel; the hips are heated to achieve a higher relaxation and resistance to pain. During the birth the mother sits on her haunches on the floor over a sheared and washed sheepskin prepared for the occasion. The person assisting the mother is in charge of cutting the umbilical cord and tie the baby's navel. This person can be her eldest daughter, her mother, her husband, one of her sisters or, less frequently, a midwife. Sometimes the delivery takes place without any assistance.

When complications arise and the placenta is not expelled, different measures are taken, such as eating herbs for inducing its discharge; massaging the hips and belly bruskly and maintaining the mother's head down for a moment; or having her blow into a bottle or bite the handle of a white knife.

A rural doctor can take part by making the appropriate prayers and making the cross with his left foot sandal three times over the mother. The latter is thought the most effective method.

The embryonary annexes must be buried in holes 1.5 m deep after being mixed with ashes. This task is carried out by the husband or the midwife. Adequate sites can be under the cooking stove or in the corrals where the family has her crops. If the embryonary annexes is not treated this way it is said that the mother can remain with a pot-belly or that the baby can suffer hernia or get sick in the stomach. It is said that this results from the annexes becoming humid when they were exposed to the open air. On the other hand they should not be burnt, as it is important that Mother Earth gets them intact to protect the child. It is believed that the strength of the child's teeth is related to the depth of the hole; the deeper the hole the longer the teeth will take to come out and the longer they will last. It is also believed that if these measures are not observed, the child could have mental problems as an adult or be indifferent to his birthplace and his mother.

### Post partum

After the delivery the mother must rest for at least a week. Besides, she must have special drinks and food for 15 days to one month. This will be discussed further below.

One week after the delivery the mother is authorized to wash herself or take a bath. Before that, she should avoid any contact with water. The bath is taken in a lukewarm infusion prepared with hot herbs. All these precautions are taken to avoid "coolness" and to induce the production of milk.

Three ailments in particular can be caught during this period if the necessary precautions are not taken: air, "inflamación" and "desmagradura".

### Air

When the mother gets up after the resting period it is advisable for her to put pieces of cotton into her ears and cover her head. This is done to avoid the entrance of air which could cause temporary or permanent deafness and general weakness.

### Inflamación

This condition is the product of a bad depuration of the body due to the "interaction" of the puerperal blood with outdoor humidity or with the heat from the cooking stove. According to local testimonies "it is the humidity that mingles with the blood and causes the bad" or "it is the blood that does not come out because of the heat of the cooking stove". If a woman gets close to water before the advisable period of restriction her eyes and her belly can become swollen and get ill of "inflamación" (swelling), the same will happen if she gets close to fire prematurely. Those who suffer from "inflamación" have their feet, hands and all their bodies swollen, get a temperature, have to stay in bed and generally die. In exceptional cases a woman can get ill of "inflamación" if she takes baths during her menstrual period.

### Desmagradura or Lastimadura

This is the local name given to uterine prolapse. To avoid it, it is recommended to use girdles for a month after the delivery. This ailment is diagnosed when strong pain appears under the ribs, nausea and vomiting are frequent; or in more serious cases, with the presence of hemorragea. To cure it, the patient goes to the rural doctor who should perform a "compostura" or "alumbriada".

This practice consists of rubbing the whole body with "coca", alumbre (alum) and alcohol; then the local doctor holds the patient with her arms crossed over her chest and exerts pressure so that her back "sounds". Finally, the patient must wear a girdle and avoid heavy duties for some days; the treatment is completed by drinking "toronjil" balm.

### Measures to ensure or induce the production of milk

When a mother produces little milk, it is assumed that she got cold in her back and in her chest during or after the delivery. For this reason, most of the corrective measures consist in warming those parts of the body, such as washing the breasts with infusions prepared with *hot *herbs or tying recently-baked hot bread to her back. Also, to treat this condition the woman must eat more and more frequently special food considered nourishing (see below).

### Care of the newborn

At birth, the baby is assisted by a relative (his grandmother, his mother's, mother in law, an elder sister or the midwife). This person cuts his umbilical cord with small scissors or with a "tiesto" (a broken piece of earthenware). The latter method is rarely practiced anymore, in older times if this element was used it was thought to foster the development of a modest and cautious personality. The navel is tied with a black and white cord, called "hilo yoque". This cord is made from sheep wool weaved to the left and it has a symbolic value; it is believed that it helps the healing of the navel. The navel must be kept dry until it falls out, and in the exceptional case in which infections or ulcerations appear it is recommended to sieve almond oil, a burnt and ground chicken feather or the spores of some fungus.

Immediately after birth, babies are given a bath with hot herbs. In some cases the habit of making them drink some special depurative preparations, as for example one or two teaspoons of almond oil or of urine of a young child, is still practiced. The mothers that observe this habit say that if this is not done the babies will later cry constantly as the result of stomachaches caused by worms that develop inside them. If the mother did not give anything to the baby immediately after birth, and it is noticed that the baby suffers stomach problems, then aromatic herbs together with black wool, tobacco and "chuspa e cacu" (the nest of *Cacicus chrysopterus*; Icteridae, Aves) should be fumigated around his craddle and his anus.

If babies are hyperactive and cannot sleep well, it is advisable to put a "churito" (the shell of local snail, *Megalobulinus *sp.) under its pillow.

### Ailments

#### Menstrual pains

Menstrual problems are considered conditions that can be treated with a great variety of medicines from the local natural pharmacopoeia coming from plants, animals, or rocks. Most women know a wide range of alternative cures.

In Additional file 1 the different herbs that are used in case of strong menstrual pains (menorrhagia) are shown. Many of them coincide with those used during the delivery and post partum period. This is because during menstruation one of the principal risks is also that of "cooling" and therefore the woman is exposed to air. It is believed that a woman in her menstrual period is capable of causing certain "evils". For example during her period she should not visit orchards or other crops as her visit could ruin the results of the harvest; she should never touch neither fruit trees, nor milk cows or ride horses. In all cases the result is a gradual weakness and, at the worst, the death of the touched animal or plant. Sexual abstinence is not considered necessary during menstruation.

#### Margaritas

In the communities under study a sexually transmitted disease called Margaritas is recognized. According to the doctors of the local hospital it is gonorrhea and occasionally syphilis. The same is considered a "chimbosa" disease, which means that if a person has got it, he favours its cure if he voluntary spreads the disease by contagion to others.

#### Sentaderas

This is the name given to both bacterial and mycotic vaginal infections. It is considered an ailment caused by coldness which enters the body. It is thought to come from the humidity or the warmth in places where women sit to rest or wash clothes. It is also thought that a woman can get this disease if she argues with somebody; the resulting anger causes the evil indirectly. As the actual origin of the disease is conceptually believed to be emotional or cultural, this kind of condition can only be diagnosed by a "rural doctor" through a "alumbriada", which is to say a "limpia"(cleansing) made with alum which is later burnt and whose ashes are interpreted by this doctor.

#### Ethnobotany

**Figure 2:**
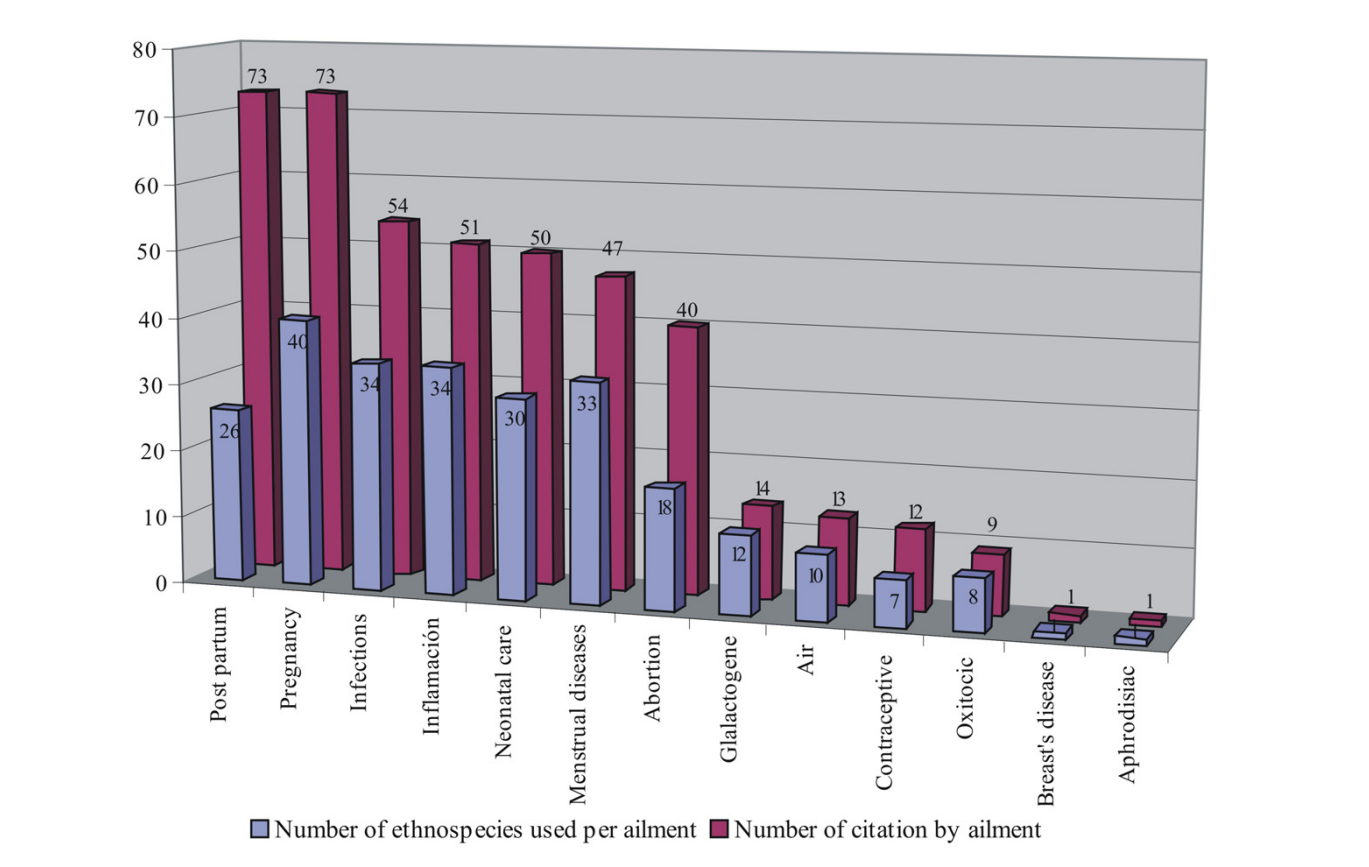
Number of ethnospecies used per ailment and number of citations for each illness.

Thirteen (13) ailments and/or different physiological states are identified and treated. In Figure 2 the number of ethnospecies used per ailment and the number of citations for each illness are shown. In relation to the part of the plant used, 55% of the reported uses correspond to the leaves and stem, 18% to reproductive organs; 11% to the whole plant; 10% to underground organs and 6% to the bark.

Taking into account the procedence of the medicinal plants, 59,61% (68) of the species used are native species (3 endemic) and the rest (46) come from another biome (10 naturalized). Gathering is the main way of obtaining them (53%) followed by cultivation (Additional file 1).

**Figure 3:**
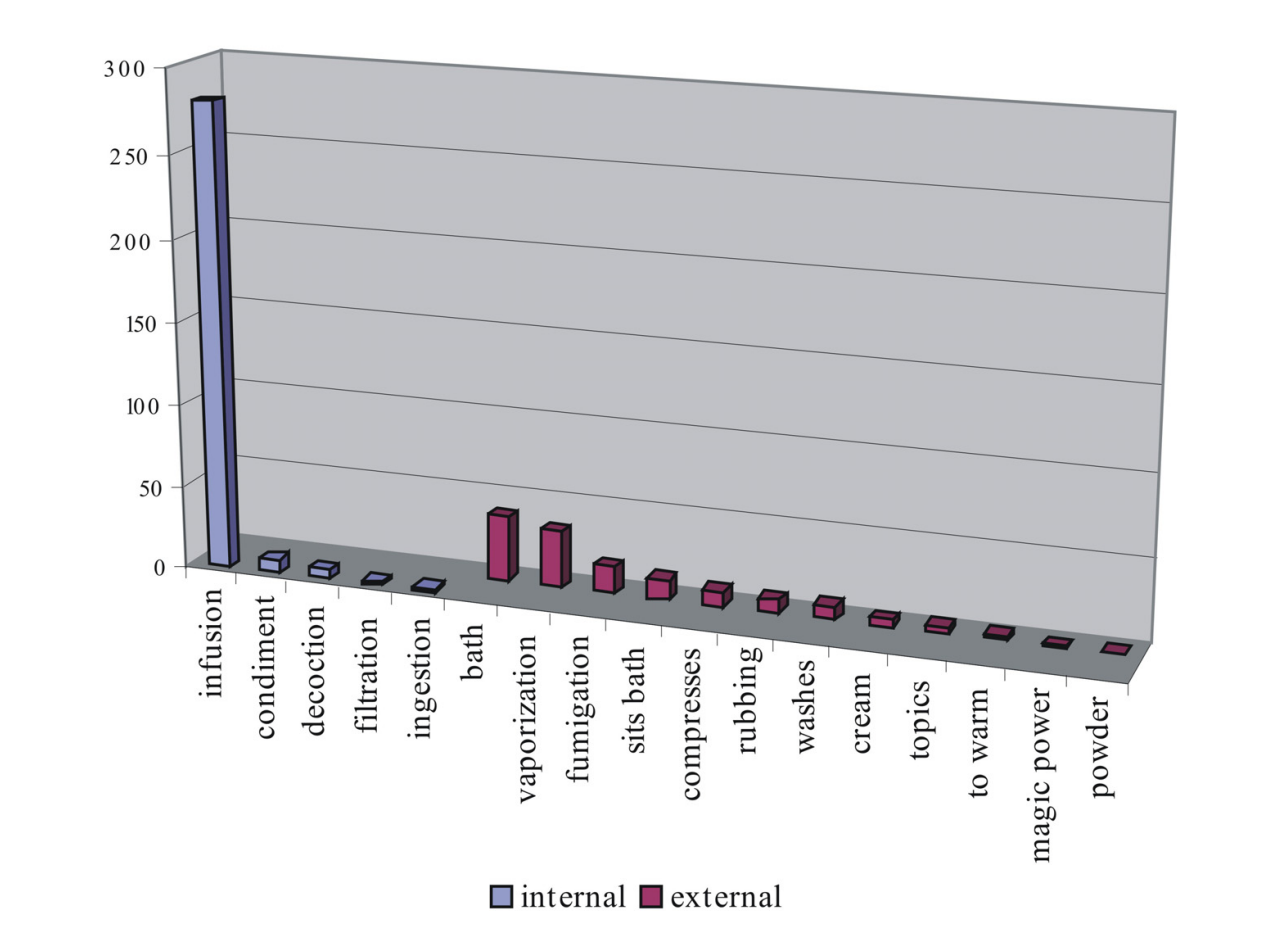
Methods of application of the medicinal plants.

In relation to the preparation, of a total of 439 registered recipes, 244 involve only one species, 101 require the mixing of 2 or more species and 94 require the addition of other ingredients, such as blood, excrements, soil, rocks, etc. With respect to their application, 140 recipes are for external use (60 with only one species, 43 two or more plants, and 37 adding elements other than plants) and 299 for internal use (184 with only one species, 43 two or more, 57 adding elements other than plants). In Fig. 3 the different methods of application are described in detail.

According to the present results all local populations use at least 108 ethnospecies for reproductive medicine, comprising 107 species of plants and 2 of mushrooms (in addition to 3 species which have been identified to genus and 2 unidentified species purchased as fragments). The species known include 42 families of flowering plants and 4 Pteridophyta families. Nine families (Asteraceae, Fabaceae, Lamiaceae, Apiaceae, Piperaceae, Poaceae, Lauraceae, Myrtaceae and Rubiaceae) comprise nearly half of the total. These medicinal plants encompass 18 pharmacological properties. Analgesic, depurative and haemostatic agents are especially common. *Peperomia fiebrigii *has been assigned 8 pharmacological properties, *Tripodanthus acutifolius *has 7, *Adiantum lorentzii *6. Five species have 5 pharmacological properties, 64 species have between 4 and 2 properties and 36 species have only one pharmacological property.

The values of relative importance (RI) vary between 12.50 and 93.75. The highest value is for *Peperomia fiebrigii*, followed by *Ruta chalepensis *and *Tripodanthus acutifolius *(81.25), *Satureja boliviana *and *S. parvifolia *(75) (see Additional file 1).

With respect to the similarities and differences in the way of use of the plants between communities, results showed that the quantity of informants by ethnospecies among all the communities have significant differences (Fcalc = 32; Ftable = 2,73; *v*_1 _= 7; *v*_2 _= 223; p = 0,01).

The same was observed with the comparison of the possible pairs of communities (Qcalc = 14.50 to 116.16; Qtable = 4,134; df = 8; p = 0.001).

In the comparison of the diversity of ailments for which each ethnospecies is used, between communities, most of them also showed significant differences. Nevertheless it was found that *Anthemis cotula *+ *Matricaria recutita*; *Petroselimun crispum*; *Ilex paraguariensis*; *Nicotiana tabacum*; *Tagetes filifolia *+ *T. minuta*; *Campyloneurum aglaolepis*; *Coffea arabica; Coronopus didymus; Erythroxylum coca*; *Schinus molle*; *Microgramma squamulosa*; *Rosa sp*. and *Salvia gilliesii *are used in a similar manner in the communities in which they were mentioned.

### Medicinal food

Twenty two (22) species were found (20 ethno-species) prescribed as curative or preventive food.

Seventeen were exotic, eleven bought and six sown, and five native, gathered. Seven are used in seasoning, nine in the preparation of infusions, four as main ingredients (guinea pumpkin, rice, corn and peanut) and two as secondary ingredients (see Additional file 1).

Due to the "warmth" generated by the baby, the use of fresh food is recommended during pregnancy. In contrast, during the post partum period, a careful selection of hot food and drinks are prescribed. Besides, hot food and drinks should be consumed for 15 days to one month. Water should not be drunk without being boiled, and it is advisable not to drink it if it is not mixed with different herbs and bee honey or roasted sugar. All food should be eaten hot or lukewarm. The more adequate dishes contain chicken meat, toasted corn, and "oregano". Beef and pork, potatoes, sugar (not roasted) and citrus fruit are some of the food considered fresh and should be avoided.

When problems arise during lactation (either scarce or no production of milk) hot food should be eaten in a larger quantity and more frequently, as it is considered to have nourishing effects. Concerning this point it can be stated that the lack of milk is explained in the region as a consequence of the mother's weakness or deficient feeding. Rice as well as peanuts are recommended when these problems appear during lactation.

### The relationship between traditional medicine and health centers

In Table 1, 27-month (Dec 1996–March 1999) hospital statistics are shown. The communities with a higher birthrate were Lipeo and Baritú with 11% and the community with the highest mortality rate for children under 1 year of age was El Condado with 12.5%. In the analysis of the degree of association between the accessibility to the Health Center, the alive/dead born babies and the assisted/non-assisted deliveries, a positive maximum association was found (R = 1) between the accessibility and the assistance during deliveries (the better the accessibility the more the assistance). In contrast, no association was found between the accessibility and the mortality of less than one-year-old dead babies (R = 0.40; t = 0.617213; p = 0.6).

**Table 1:** Population growth and hospitals/health centers

	Population increase in 27 month (Number of births)	Total population (one-year increase)	Deliveries in institutions (hospitals/health centers)	Children who died before 1 year of age
El Condado	6% (24)	389 (2,6%)	58%	3 (12,5%)
La Misión	7.6% (34)	443 (3%)	70%	2 (5,8%)
Los Toldos	9% (39)	431 (4%)	87%	0%
Arazay	6.4% (25)	388 (2.9%)	76%	1 (4%)
Lipeo-Baritú	11% (22)	201 (4.8%)	13%	1 (4,5%)

Official data for the term December 1996/March 1999

## Discussion and conclusions

The observations made in the area agree well with those described for other Andean communities. Taking into account the moment in which plants considered contraceptive are used during the female menstrual cycle it is difficult to define whether their use corrects delays (amenorrhea) or causes abortion, which coincides with that observed for Spanish New Mexicans [16].

Even though both the religious views in the area and public health agents prohibit abortion, given the local preconceptions about the procedence of pregnancy, those who used abortive plants do not suffer personal or communal conflicts, because if pregnancy is interrupted it is because the fetus is not human. Similar cultural interpretations were found for possible causes of pregnancy in neighbouring communities which dwell the Valles Calchaquíes [18,20].

The effect of the embryo on the mother's temperature balance was cited in numerous cases in Andean communities and the main preventive or regulatory measures in all of them are observed in the diet. The magical and/or supernatural conditions that anticipate the delivery have been registered in other neighbouring communities as well; the aromatic herbs, the sheared sheep skin, the "hilo yoque" to tie the navel, the special tools traditionally used to cut the umbilical cord, the way of inducing the placenta and annexes to discharge and the measures taken during the post partum period have been registered and explained in numerous studies [5,15,18-22,24].

It is possible that the habit of burying the placenta as a propitiatory action for the good personal and physical development of the newborn have the same connotation as the habit of burying the rest of cattle during branding or the offerings buried the first of August to honor the Pachamama: to propitiate the success and fecundity of the newborn [45]. Mariscotti [46], when explaining this type of offerings to the Pachamama (ctonic Andean Goddess), suggested that they are surviving practices of the ritual sacrifice in her honor.

In all cases the mother's sudden cooling at the moment of delivering the baby makes her susceptible to getting air and as a result to become ill.

In what is related to air and the "aicadura", we have found the same conceptual basis but with different names in studies made in Creole communities with Quechuan influence in neighbouring regions, in which they are referred to as "sobreparto" and air [2,3]. In both cases (air and "aicadura") the person resorts to the "rural doctor" who performs secret cures, such as "limpias" and "alumbriadas" in which plants clearly play a secondary role in the therapy (more details in Hilgert [29] and Amodio [47]).

According to the local notion the body cooling or overheating affects the function of the corporal humors, thus menorrhagia, menstrual hemorrhaging, placental retention and other post-partum disorders are explained by an alteration in the blood; these kinds of ideas are common in different cultures [2,3,5,14,15,19,48]. The templary imbalance constantly appears as a "producer" of ailments and practically all measures taken in these cases are preventive ones. Due to the initial objectives we posed and the methodology we have used in this study, we are not able to discuss this topic in depth (although we consider it of importance). To be able to do so, deeper studies should be made in ethnomedicine, paying special attention to the historic antagonism with respect to this matter that is shown in the available literature [3,7,49,50].

In relation to the medicinal plants, the most cited species was *Origanum × appli*; *Peperomia fiebrigii *was found to have the highest number of assigned uses and the highest relative importance. These species together with *Tripodanthus acutifolius, Satureja boliviana *and *S. parvifolia *were the most important both in the number of reports, the number of treated conditions as well as in their relative importance. In contrast, it was observed that the ethnospecies with the highest value of use (RI) which are used in a similar way in more communities are, in decreasing order: *Erythroxylum coca, Anthemis cotula *+ *Matricaria recutita, Schinus molle *and *Coronopus didymus*.

In relation to the special food recommended during the gestation and the post partum period, the concept of hot and cold and the type of food chosen in different Andean regions are very similar to the data based on the communications analysed in the present study [15,23,24]. As most of the species used in this way do not belong to the Andean prehispanic repertoire, it is highly possible that the medicinal culinary tradition is a recreation that resulted from the combination of both cultural currents.

This would reinforce the proposed idea that some of the present medicines have been introduced as foodstuffs [28].

Pieroni [25] states that for the feeding tradition of the centre of Italy there are three types of food, their local value much more founded in medicine than in feeding: infusions or aromatized grappa, seasonings with digestive or carminative functions and "cleansing" wild greens. In the local reproductive medicine, the first two are undoubtedly represented, however it is thought that the addition of feeding supplements constitutes an important function, which is not explicit in the local value.

The reproductive ailments are resolved almost exclusively within the family environment. The role of midwives and physicians is very limited by the privacy with which these matters are dealt with. It is probable that a part of the explanation of the low consensus found among informants and among communities may be attributed to family secrecy.

Finerman [15] observed a similar situation, where domestic medicine prevails over institutional medicine, in Quechuan communities in the south of Ecuador. There, although mothers accept that deliveries in hospitals are safer, they refuse to go to them. The author proposes that this is due in part to the fact that in hospitals many of the practices firmly rooted in the local culture cannot be carried out (such as a 40-day rest) and also to the lack of privacy which generates stress and anxiety in the delivering mother.

In nearby communities, Bianchetti [19] explains that the reluctance to go to the health centers is base on the fact that the reproductive process in the regional culture is widely linked to magical-empirical principles that are far from the scientific concept of occidental medicine. He further suggests an inadequate approach on behalf of the formal medicine participants, to the point of mentioning that in the Argentine Altiplane mothers are obliged to give birth in institutions, and if they do not do so, birth certificates are not given to them. The latter has not been observed in our study area.

In the area under study it is probable that the reluctance, especially of the families coming from far away places, is based on the absence of an effective link with the physician and on the lack of a lodging site for the delivering mother and a relative before and after the delivery.

Furthermore, a better communication and understanding of certain simple cultural matters by the hospital staff would also increase the number of institutional deliveries. For example, if the embryonary annexes were given to relatives and the food of the mother was prepared with the ingredients and according to the traditional recipes and prescriptions.

## Competing interests

The author(s) declare that they have no competing interests.

## Authors' contributions

The first author made the fieldwork. Both authors share the data analysis and compilation on this manuscript.

## Supplementary Material

Additional file 1 Medicinal plants and uses. The data lists the plants species used in reproductive medicine, their botanical and family name, voucher specimen code, folk name, provenience of the plant material, parts of the plant used, claimed medicinal use and modes of administration, hot cold syndrome classification, reports number and Relative Importance Index. Click here for file
