# Supplementary material for: Reproductive medicine in northwest Argentina: traditional and institutional systems
Source: J Ethnobiol Ethnomed. 2007 May 2;3:19. doi: 10.1186/1746-4269-3-19 (PMC1876447; doi:10.1186/1746-4269-3-19)
Supplement: Additional file 1 — Medicinal plants and uses. The data lists the plants species used in reproductive medicine, their botanical and family name, voucher specimen code, folk name, provenience of the plant material, parts of the plant used, claimed medicinal use and modes of administration, hot cold syndrome classification, reports number and Relative Importance Index. [file 1746-4269-3-19-S1.pdf]

Table 1. Medicinal plants and uses.

Botanical taxon (taxa), family and voucher specimen code(s)	Folk name(s)	Provenience of the plant material	Part (s) used	Claimed medicinal use	Administration	Hot cold syndrome	N° reports	RI
Peperomia fiebrigii C. DC., PIPERACEAE (985, 2036, 2188, 2345, 2450, 2522)	siemprevida grande, siemprevida, siempreviva, congojita	g	plant	neo.<br>ab.; gl.; infl.; mens.; prg.; pstp.	bath<br>infusion, vaporization	very hot	22	93.75
Ruta chalepensis L., RUTACEAE (1399, 2262, 2434)	ruda	c	aerial parts	ab.; air; mens.<br>inf. ; mens; infl.; prg.; pstp.	infusion<br>bath, fumigation, vaporization	hot-temperate	10	81.25
Tripodanthus acutifolius (Ruiz & Pav.) Tiegh., LORANTHACEAE (1901, 1093, 1096, 1413, 2248)	corpo	g	flower	ab.; ctp.; mens.; neo.; prg.; pstp.	infusion	hot- very hot	20	81.25
Satureja boliviana (Benth.) Briq., LAMIACEAE (2335, 2443, 2528); *Satureja parvifolia* (Phil.) Epling, LAMIACEAE (2269, 2166)	muña, muña muña	g	leaves<br><br>aerial parts	air.; neo.<br><br>inf.; infl.; mens.; prg.; pstp.	fumigation or vaporization<br>infusion*, condiment*, vaporization, bath	hot	7	75.00
Tanacetum parthenium (L.) Sch. Bip., ASTERACEAE (1479, 943)	ajenco grande, ajenco	c	aerial parts	ab.; mens.	infusion		2	62.50
Peperomia tetraphylla (G. Forst.) Hook. & Arn., PIPERACEAE (1426)	siempreviva chica	g	plant	gl.; inf.; infl.; neo.; plc.; prg.	infusion		7	62.50
Rosmarinus officinalis L., LAMIACEAE (2478)	romero	c	leaves	air; inf.; infl.; neo.; prg.; pstp.	fumigation, vaporization, rubbing cream	hot	8	62.50
Erythroxylum coca Lam. var. *coca*, ERYTHROXYLACEAE (2108)	coca	b	leaves	air<br>neo.<br>inf.; plc.; prg.	fumigation<br>rubbing<br>rubbing,	hot	11	62.50

					infusion, fumigation			
Anthemis cotula L., ASTERACEAE (1410); *Matricaria recutita* L., ASTERACEAE (2442, 2438, 2479, 2227)	manzanilla	gc	flower<br>aerial parts<br>flower<br>flower, aerial parts<br>flower	inf.<br>infl.<br>mens.<br>neo.<br>prg.	rubbing<br>sits bath<br>infusion<br>bath, rubbing<br>cream, infusion	hot	12	56.25
Petroselinum crispum (Mill.) A.W. Hill, APIACEAE (1959, 2547, 2245)	perejil	c	aerial parts<br>aerial parts	infl.; prg.; pstp.<br>ab.	infusion, condiment*<br>topics, infusion		7	56.25
Adiantum lorentzii Hieron., PTERIDACEAE (1457)	culandrillo	g	plant	mens.<br>prg.<br>pstp.	vaporization, infusion<br>vaporization, washes, infusion<br>compresses, infusion	hot	22	56.25
Acacia aroma Gill. ex Hook. & Arn., FABACEAE (2288, 2539, 2567)	bais, tusca	g	bark	ab.<br>infl.<br>prg.; pstp.	infusion<br>to warm<br>bath	cold-temperate	5	50.00
Coronopus didymus (L.) Sm., BRASSICACEAE (2295, 1987, 1065, 944, 957)	quimpy	g	plant	infl.; mens.; prg.<br>neo.	bath, infusion<br>bath	cold	9	50.00
Ilex paraguariensis A. St. Hill., AQUIFOLIACEAE (1565)	yerba, yerba mate	mp	leaves	air<br>gl.; neo.; pstp.	fumigation<br>infusion*	hot	7	50.00
Microgramma squamulosa (Kaulf.) de la Sota, POLYPODIACEAE (2173, 2346, 2452, 2525)	pori pori, polipor	g	plant	aphro.; inf.; infl. prg.	infusion		10	50.00
Rosa sp., ROSACEAE (1444, 1445, 2261, 2572)	rosa, r. mosqueta, r. remedio	c	flower	inf.; mens.; neo.; prg.	infusion, bath	cold	5	50.00
Salvia gilliesii Benth.,	salvia gateadora	g	aerial	air	fumigation		5	50.00

LAMIACEAE (2321)			parts	inl.<br><br>prg.; pstp.	infusion and sits bath<br>infusion			
Origanum x appli (Domin) Boros, LAMIACEAE (1448, 2242)	orégano	c	aerial parts	mens.; prg.; pstp.	infusion, condiment*	hot	30	50.00
Nicotiana tabacum L., SOLANACEAE (1474, 2228)	tabaco	c-mp	leaves	air; pstp.<br>neo.; prg.	bath<br>fumigation	temperate	4	43.75
Not identified, FUNGI (1489, 1490, 1491, 1492, 1493, 1494, 1495, 2176, 2483, 2484)	oreja de palo	g	aerial parts	ab.; ctp.; inf.; mens.	infusion		5	43.75
Sambucus nigra L. subsp. *peruviana* (Kunth) R. Bolli, CAPRIFOLIACEAE (2142, 1075)	mololo	g	flower<br>flower and leaves	inf.; prg.; mens.<br>neo.	infusion<br>bath	hot<br><br>cold	13	43.75
Schinus molle L., ANACARDIACEAE (2540, 2278)	molle	gc	leaves	air<br>neo.<br>inf.; infl.	fumigation<br>washes<br>vaporization, compresses	hot	6	43.75
Tagetes filifolia Lag., ASTERACEAE ((1061, 1985); *Tagetes minuta* L., ASTERACEAE (2180)	anís del campo	g	aerial parts	inf.; neo.; prg.; pstp.	infusion*		6	43.75
Campyloneurum aglaolepis (Alston) de la Sota, POLYPODIACEAE (1427)	polipor, pori pori	g	plant<br>plant	inf.<br>mens.; prg.	infusion<br>infusion		4	43.75
C*inchona ledgeriana* Moens., RUBIACEAE (2472)	quina castilla	b	bark<br><br>bark<br>leaves and bark	infl.<br><br>neo.<br>prg.	cream, infusion<br>washes<br>infusion		5	43.75
Coffea arabica L., RUBIACEAE (1641)	café	mp	seeds	mens. ; prg.; pstp.	infusion*	hot	6	43.75
Cortaderia selloana (Schult. & Schult. f.) Asch. & Graebn., POACEAE (1772, 2440)	cortadera	g	roots	mens.<br>prg. ; pstp.	bath, infusion<br>infusion		6	43.75

Baccharis trimera (Less.) DC., ASTERACEAE (2277, 2344, 2501)	carqueja	g	aerial parts<br>leaves, aerial parts	mens.; neo.; prg.<br>pstp.	infusion<br>condiment*<br>infusion	cold	5	37.50
Arundo donax L., POACEAE (2577)	caña hueca, caña hueca castilla	c	roots	ab.; ctp.; plc.	infusion		3	37.50
Blumembachia sp., LOASACEAE (2599)	itapalla del cerro	gc	flower	infl.; mens.; pstp.	infusion	temperate, hot	3	37.50
Caiophora lateritia (Hook.) Koltzch, LOASACEAE (2225)	itapalla	g	flower	ab.; mens.; prg.	infusion	hot-very hot	3	37.50
Cuminum cyminum L., APIACEAE (1593)	comino	b	seeds	gl.; pstp.<br>mens.	condiment*<br>infusion	hot	3	37.50
Myroxylon peruiferum L.f., FABACEAE (1632, 2377)	quina del campo, quina	g	bark	infl.<br>neo.<br>prg.	vaporization, to warm<br>bath<br>bath, infusion	cold	5	37.50
Pavonia sepium St. Hil. , MALVACEAE (1099)	abrojo	g	roots	infl.; mens.; prg.	infusion	cold	3	37.50
Plantago australis Lam. subsp. *australis*, PLANTAGINACEAE (2534)	llantén, llantén blanco	g	roots<br>leaves	inf.; infl.<br>prg.	infusion and compresses<br>infusion	cold	3	37.50
Smilax campestris Griseb., SMILACACEAE (2320, 2458)	zarzaparilla	g	roots	inf.; infl.; mens.	infusion, decoctions, bath		7	37.50
Syzygium aromaticum (L.) Merr. & Perry, MYRTACEAE (1580)	clavo	b	sprouts	mens.; prg.	infusion*, condiment*	hot	2	31.25
Equisetum bogotense H.B.K., EQUISETACEAE (1394)	cola de caballo chica	g	plant	mens.; prg.; pstp.	vaporization, decoctions		4	31.25
Malva parviflora L., MALVACEAE (2490, 2557)	malva	g	aerial parts	infl.; neo.	bath, to warm, vaporization	cold	3	31.25
Persea americana Mill.,	palta anisada,	c	seeds	inf.	sits bath		3	31.25

LAURACEAE (1650, 2073, 2074)	palta		leaves	mens.	infusion			
Anadenanthera colubrina (Vell.) Bernan var. *cebil* (Griseb.) Alts., FABACEAE (2398)	cebil	g	bark	ab.; inf.	infusion		5	25.00
Apium graveolens L., APIACEAE (2436)	apio, apio de huerta	c	aerial parts	infl.; pstp.	infusion, ingestion (secondary ingredient)*		2	25.00
Artemisia absinthium L., ASTERACEAE (2433)	ajenco	c	leaves	ab.; plc.	infusion		3	25.00
Celtis iguanaea (Jac.) Sarg., CELTIDACEAE (2148)	tala	g	aerial parts<br><br>leaves	infl.<br><br>mens.	sits bath<br>infusion		2	25.00
Cinnamomum porphyrium (Griseb.) Kosterm., LAURACEAE (2022, 983)	laurel	g	leaves<br>bark	neo<br>prg.	bath<br>infusion	cold	4	25.00
Cinnamomum zeylanicum Blume, LAURACEAE (2330)	canela	b	bark	mens. ; prg.	infusion, condiment*	hot	3	25.00
Citrus aurantifolia (Christm.) Sw., RUTACEAE (2143, 2388)	lima	c	leaves	gl.<br>neo.	infusion		2	25.00
Citrus sinensis (L.) Osbeck, RUTACEAE (1591, 2053)	naranja	c	epicarp	air<br>gl.	fumigation<br>infusion	hot	3	25.00
Cucurbita maxima Duchesne subsp. *maxima*, CUCURBITACEAE (1573)	guinea zapallo	c	fruits	gl.<br><br><br>plc.	ingestion (principal ingredient)*<br>infusion		2	25.00
Equisetum giganteum L., EQUISETACEAE (1617)	cola de caballo	g	plant	mens.; prg.	vaporization, decoctions		4	25.00
Eugenia uniflora L., MYRTACEAE (1101, 1503)	arrayán	g	leaves	infl.; mens.	infusion		2	25.00
Eupatorium bupleurifolium DC., ASTERACEAE (2172, 2025)	prementina	g	aerial parts	mens.; plc.	vaporization, infusion	hot	3	25.00

Galium latoramosum Clos, RUBIACEAE (1443); *Galium lilloi* Hicken, RUBIACEAE (2389)	chipi chape	g	Plant	ab.; ctp.	infusion		2	25.00
Krameria lappacea (Dombey) Burdet & Simpson, KRAMERIACEAE (2300)	chipichape	g	aerial parts	ab.; ctp.	infusion		2	25.00
Lepechinia vesiculosa (Benth.) Epling, LAMIACEAE (1899)	salvia grande	g	aerial parts	inf.; prg.	sits bath, vaporization	hot	3	25.00
Macfadyena unguis-cati (L.) A.H. Gentry, BIGNONIACEAE (2015, 2192)	uña de gato, garra de gato	g	roots<br>plant	gl.<br>inf.	infusion<br>infusion, bath, vaporization		5	25.00
Maytenus cuezzoi Leg., CELASTRACEAE (2565)	lloque	g	leaves	inf.; infl.	vaporization, infusion	hot	3	25.00
Mentha x piperita L. var. *citrata* (Ehrh.) Briq., LAMIACEAE (1481)	menta	gc	leaves	neo.; pstp.	infusion		2	25.00
Not identified	livi livi	g	aerial parts	inf.; infl.	vaporization	hot	4	25.00
Ocimun basilicum L., LAMIACEAE (1574, 2258)	albahaca, albahaca morada	c	aerial parts	prg.; pstp.	infusion		2	25.00
Peperomia alata Ruiz & Pav., PIPERACEAE (2391)	anís	g	plant	inf.; prg.	infusion		2	25.00
Peperomia theodori Trel., PIPERACEAE (1105)	siempreviva	g	plant	mens.; prg.	infusion		2	25.00
Petiveria alliaceae L. var. *alliaceae*, PHYTOLACCACEAE (2383)	calaschi	g	roots	bre.; mens.	washes		2	25.00
Pimpinella anisum L., APIACEAE	anís de pan	b	seeds	gl.; neo.	infusion*		2	25.00
Rhipidocladum racemiflorum (Steud.) McClure, (POACEAE) 2367	caña hueca	g	roots	ab.; ctp.	infusion		2	25.00
Theobroma cacao L., STERCULIACEAE	chocolate	mp	seeds	mens.; prg.	infusion*	hot	3	25.00

Verbena litoralis Kunth, VERBENACEAE (1485, 2499)	verbena	g	aerial parts	air neo.	infusion bath	cold	2	25.00
Eucalyptus camaldulensis Dehnh., MYRTACEAE (1845)	eucaliptus	c	leaves	inf.; infl.	vaporization	hot	2	18.75
Lactuca sativa L., ASTERACEAE (1569)	lechuga	c	roots	prg.; pstp.	infusion		2	18.75
Mimosa debilis H.B.K. ex Willd., FABACEAE (947)	celosita grande, celosita hoja ancha	g	roots	inf.; infl.	infusion		2	18.75
Mimosa polycarpa Kunth. var *subandina* Barneby, FABACEAE (2220)	celosita, celosita de hoja menudita, celosita chica	g	roots	inf.; infl.	infusion		4	18.75
Buddleja brasiliensis Jacq. ex Spreng., BUDDLEJACEAE (2182, 2493); *Buddleja tucumanensis* Griseb., BUDDLEJACEAE (2570)	san juan c`ora	g	aerial parts	inf.	vaporization	hot	2	18.75
Jacaranda mimosifolia D. Don, BIGNONIACEAE (2397)	tarco	g	leaves	inf.	compresses		2	18.75
Piper aduncum L. var. *aduncum,* PIPERACEAE (2336)	matico	g	leavess	inf.	sits bath		2	18.75
Prunus amygdalus Batsch., ROSACEAE	almendras	mp	oil	neo.	topics, infusion		2	18.75
Senecio crepidifolius DC., ASTERACEAE (2603, 2500)	árnica	g	aerial parts	infl.	sits bath, compresses		2	18.75
Urera caracasana (Jacq.) Gaudich. ex Griseb., URTICACEAE (2394)	itapalla grande, orteguilla	g	roots	inf.	filtration, infusion		2	18.75
Acacia macracantha Humb. & Bonpl. ex Willd., FABACEAE (2581)	tusca	g	bark	infl.	sits bath		1	12.50
Adiantopsis chlorophylla (Sw.) Fée, PTERIDACEAE (993); *Hypolepis repens* (L.) C. Presl, DENNSTAEDTIACEAE (960)	ala e cuervo	g	leaves	mens.	infusion		2	12.50

Aloysia polystachya (Griseb.) Mold., VERBENACEAE (1449)	burrito	gc	aerial parts	plc.	infusion		1	12.50
Amaranthus quitensis Kunth, AMARANTHACEAE (2393)	aroma	gc	leaves and seeds	pstp.	infusion		1	12.50
Anredera cordifolia (Tenore) Steen., BASELLACEAE (2256, 2537)	papa santa, hierba santa, santa lucía, santa maría	c	leaves	neo.	bath	cold	2	12.50
Arachis hypogaea L., FABACEAE (2421, 2422)	maní	c	seeds	gl.	ingestion (principal ingredient)*		1	12.50
Azorella compacta Phil., APIACEAE (1044)	yareta	gc	aerial parts	ctp.	infusion		1	12.50
Baccharis coridifolia DC., ASTERACEAE (2157)	romerillo	g	leaves and aerial parts	ab.	infusion	very hot, hot	3	12.50
Bocconia integrifolia Humb. & Bonpl., PAPAVERACEAE (1633, 2541)	mil hombres	g	roots	infl.	sits bath		1	12.50
Brassica sp., BRASSICACEAE	mostaza	mp	seeds	infl.	cream	hot	1	12.50
Camellia sinensis (L.) Kuntze, TEACEAE	té	mp	leaves	pstp.	infusion*		1	12.50
Erythrina falcata Benth., FABACEAE	ceiba	g	Leaves and bark	gl.	infusion		1	12.50
Gochnatia palosanto Cab., ASTERACEAE (1619)	palo santo	b	bark	infl.	compresses		1	12.50
Juglans regia L., JUGLANDACEAE	nogal castillo	b	bark	infl.	sits bath		1	12.50
Manihot esculenta Crantz, EUPHORBIACEAE (1616)	mandioca	mp	rizome	inf.	cream		1	12.50
Melissa officinalis L., LAMIACEAE (1981)	toronjil	gc	aerial parts	neo.	infusion		1	12.50
Myristica fragrans Houtt., MYRISTICACEAE (2104)	nuez moscada	b	seeds	infl.	compresses, condiment*		1	12.50
Not identified	llavitica, barba	g	plant	ab.	filtration	hot	1	12.50

	de los churquis							
Not identified, FUNGI (2041)	polvillo, humerita	g	aerial parts	neo.	topics		1	12.50
Oreopanax kuntzei Harms, ARALIACEAE (1375)	higuerilla	g	bark	infl.	magic powers		1	12.50
Oryza sativa L., (1582)	arroz	b	seeds	pstp.	ingestion (principal ingredient)*		1	12.50
Peumus boldus Molina, LAMIACEAE	boldo	mp	leaves	plc.	infusion*		1	12.50
Prosopis nigra (Griseb.) Hieron. var. *nigra*, FABACEAE (1038)	algarroba	b	roots	inf.	bath, infusion		2	12.50
Rhipsalis lorentziana Griseb., CACTACEAE (1509)	huasca huasca, peinquillita	g	plant	neo.	washes	cold	1	12.50
Sebastiania anisandra (Griseb.) Lillo., EUPHORBIACEAE (956, 1079, 1412)	lecherón	g	aerial parts	gl.	washes		1	12.50
Senecio cremeiflorus Mattf., ASTERACEAE (1442, 2158)	lampazo	g	leaves	inf.	compresses		1	12.50
Senna crassiramea (Benth.) H.S. Irwin & Barneby, FABACEAE (2466)	sumalagua	gc	aerial parts	inf.	infusion		1	12.50
Sida rhombifolia L., MALVACEAE (2505)	afata	g	plant	neo.	bath		1	12.50
Tanacetum vulgare L., ASTERACEAE (2260)	Santa María	c	flower, leaves and flower, aerial parts	ab.	infusion		8	12.50
Valeriana officinalis L., VALERIANACEAE	valeriana	mp	roots	mens.	infusion		1	12.50
Zea mays L., POACEAE (2423)	maíz	c	stigma	prg.	ingestion (principal ingredient)*		1	12.50

Stipulated references used:

g: gathered from the wild; c: cultivated; b: bought in market/ shops; mp: bought -manufactured product-; gp: gathered or purchase; gc: gathered or cultivated; c-mp: cultivated or bought -manufactured product-. ab.: to induce abortion; aphro.: aphrodisiac; air; bre.: breast's disease; ctp: contraceptive; gl: galactogene; inf.: infections (margaritas, sentaderas); infl.: *inflamación*, hernia and prolapse; mens. menstrual diseases (dysmenorrhoea, menorrhagia, amenorrhagia); neo.: neonatal and paediatrics care; plc.: to expel the placenta and as protective post partum; prg.: used in pregnancy and facilitate birth; pstp.: post partum pains and diseases.

* Medicinal food use

RI: Relative importance (Rel PH + Rel BS) / 2) * 100. Rel BS: relative number of body systems treated (normalized to maximum value of 1); Rel PH: Relative number of pharmacological properties (normalized to maximum value of 1).
